# On Toxic Polyneuritis of the Motor Type

**Published:** 1915-03

**Authors:** J. Michell Clarke

**Affiliations:** Joint Professor of Medicine, University of Bristol, and Physician to Bristol General Hospital


### ON TOXIC POLYNEURITIS OF THE MOTOR TYPE.

BY

J. MICHELL CLARKE, M.A., M.D., LL.D., F.R.C.P.,

*Joint Professor of Medicine, University of Bristol, and Physician to Bristol General Hospital.*

---

IN the following cases of multiple neuritis objective disturbances of sensation were absent, and though there was a certain amount of pain, in one case considerable pain, the symptoms were essentially those of motor paralysis. Instances of this type of neuritis, excluding, of course, those of single nerves, are not at all common. The great majority of cases of multiple neuritis are those of alcoholic neuritis, in which sensory disturbances form one of the most striking clinical features. In the early stages of the motor type of polyneuritis, when the disease is spreading and invading successive groups of muscles, the difficulty of diagnosis from an ascending paralysis of spinal cord origin, and also from certain cases of anterior poliomyelitis may be difficult. Moreover, many of the most characteristic features of multiple neuritis, as seen in the common form due to alcohol, are absent or inconspicuous. Thus in alcoholic neuritis women are far more often affected than men, and women especially between forty and fifty years of age. The legs are involved first, and in the majority of cases the arms escape or are comparatively slightly affected, whilst pains, tenderness of nerves and muscles, and peripheral loss of cutaneous sensibility are prominent, and these symptoms, together with certain other features, especially the mental changes, which form the well-known Korsakoff's syndrome, and the cardiac weakness, due to the action of alcohol on the heart-muscle, all contribute to make up a characteristic and striking clinical picture. Again, in acute toxic polyneuritis the cause is generally obscure, and often there is no clue to its exact nature.

Except in those cases which follow influenza, or some such infective disease, the onset is insidious, may take place during apparent good health, and the absence of constitutional disturbance seems to negative the idea of a microbic infection as the cause.

During the first few days of a case of toxic polyneuritis of the motor type the resemblance to ascending or Landry's paralysis is close, so that one may have to postpone decision as to the exact nature of the illness, and may have considerable anxiety as to the event, owing to the gravity of the prognosis in the latter disease.

In the majority of cases, however, of polyneuritis the onset and progress of the paralysis is slower than in a typical case of Landry's paralysis, and in the course of the second or third week, if not at the onset, the paralysis begins to be definitely greater at the periphery of the limbs, if not in all four extremities at first, yet either in the arms or in the legs, and to predominate in the extensors, whilst alterations in the normal electrical reactions of the most affected muscles begin to be evident. These changes in electrical reactions precede the wasting, which, indeed, in some cases is not very marked throughout the illness.

The diagnosis from acute anterior poliomyelitis is not so difficult, as in the great majority the sufferers from this disease are much younger than those from acute toxic polyneuritis, and if in exceptional cases the age cannot be taken into account in distinguishing between the two affections, there are the decided signs of constitutional disturbance at the onset of acute poliomyelitis, the characteristic pains on passive movements of the joints, the absence of such symmetrical paralysis, the escape of a muscle or group of muscles in the otherwise paralysed limb, the recovery of some muscles soon after the onset, and the frequent presence of a large lymphocytosis in the cerebro-spinal fluid.

The first case I report is one of the senile type of the disease, and corresponds to the usual descriptions of this form in its insidious course, and absence of pain or other sensory disturbance; but although sensory symptoms are usually comparatively inconspicuous, they are rarely so completely absent as in this instance, in which also there was not the marked arterio-sclerosis which is generally associated with senile neuritis. The diagnosis presented no difficulty, as the signs of polyneuritis were characteristic by the time the patient came under observation.

This patient was an emaciated man aged 72. The family history was good. He had never had any illness of any kind, had always been strong, healthy and temperate, and worked very hard as a blacksmith.

Six weeks previous to admission he felt quite well on getting up, but on going to work noticed that his right leg dragged; he was, however, able to stay at work without inconvenience during the whole of that day, but on going to bed he had to be helped upstairs, as both legs were so weak that he had great difficulty in getting upstairs even with help. He had a good night, but on waking the next morning he found the weakness had so increased in the legs that he could not stand, and had spread to both arms, so that he could not raise himself in bed. There was no constitutional disturbance of any kind, and no pain. The limbs felt "dead" and numb. Since this time he has remained *in statu quo*, confined to bed and practically helpless. Sphincters, speech, swallowing and special senses unaffected.

On admission he was very helpless and unable to move himself in bed. Tongue clean. Pulse 104. Respiration 24. Temperature subnormal. The few teeth remaining were bad. No blue line on gums. There was a moderate degree of atheroma of the vessels. Both legs were powerless except for slight movements at left hip. The feet were dropped, and toes flexed. There was also double wrist-drop, the fingers were slightly flexed and could not be extended. With great effort the right arm could be moved feebly at the shoulder, and very weak movements of the left were possible at shoulder and elbow, with feeble flexion of wrist. He could not feed himself nor use the hands for any purpose. The head and neck could be moved freely. The muscles of all limbs were wasted, especially the extensors of wrist, fingers, ankles and toes.

Diaphragm and intercostal muscles acted well.

Electrical reactions. Loss of reaction to Faradic current in extensors of left wrist and fingers, interossii of right hand, anterior tibials and peronei muscles of both legs ; very feeble reaction to strong current in long extensors of right wrist and fingers and muscles of thumbs. The flexor muscles responded but feebly in arms ; calf muscles also reacted feebly. All muscles reacted to the constant current, but the extensors gave a slow wave, and A C C = K C C . In the flexors the response was normal.

There was no affection of sensation to pain, light touch, heat or cold. Sense of position and of movement of limbs unaffected. No muscular tenderness.

There was a slight tremor of the left arm when movement was attempted. No cramps or spasms.

*Reflexes.*—Deep reflexes absent. Superficial : abdomina and cremasteric present, plantar absent.

The paralysis was entirely flaccid in type.

Cranial nerves unaffected. Pupils equal, acted well to light and accommodation. Optic discs normal. Thoracic and abdominal organs normal. Urine normal. Sphincters unaffected.

He remained in the hospital one week, his condition being unchanged, and then refused to stay any longer.

In the next case the diagnosis was for some little time doubtful, owing to the completeness of the paraplegia, and to the extensors not being predominantly affected until late after the onset. For a time also the electrical reactions were unaltered. Wasting of the muscles did not occur for a long time, and was never conspicuous throughout the illness. In fact, the slightness of the wasting in muscles which remained paralysed for months was remarkable. The pains in the cervical spine soon after the onset also introduced a doubtful element in diagnosis. The time taken for recovery, a little over twelve months, shows what is to be expected as to duration in a severe case of this type, and also that even if little progress is made for six months this need not preclude complete recovery. The case, indeed, illustrates that recovery may be perfect, if due care is taken throughout the illness in the prevention of contractures and of unfavourable position of the limbs ; so favourable a result is hardly seen in any other form of paralysis. Throughout the illness there was no affection of sensation, beyond at one time a doubtful and partial anæsthesia to light touch on the outer sides of the feet. Efforts were made to find out the nature and source of the toxin, but without success. Examination of the blood and cerebro-spinal fluid and bacteriological examinations of the fauces and urine gave no clue to it.

John B., aged 34, a joiner, had always enjoyed good health, except for a mild attack of jaundice when in the Argentine five years previously. He was in a very healthy district, and never had malaria. He had never had syphilis, and had always been very temperate. Eight weeks previously he had a sebaceous cyst removed, which left a discharging sore for three weeks and then healed well. He had no sore throat. On September 14th, 1913, he had cramp in the muscles of the right leg, followed next day by stiffness in both legs, then by weakness. The stiffness and weakness gradually increased until the 18th, when his feet tingled and became so numb that he could not tell their position, and consequently found great difficulty in walking. On the 19th he could not walk to his work, but managed to get there on his cycle. On the 20th he found he was unable to walk without support, as he lost his balance, and was unable to tell the position of his legs unless he looked at them. On this day numbness and tingling, with weakness, also appeared in both hands. On the 23rd he felt a feeling like a " lump of lead " in the abdomen. From this date the weakness of the limbs steadily increased, and he was confined to bed until his admission to hospital on October 2nd.

On admission he was well nourished and healthy-looking. The pulse was 88, the respiration, tongue and temperature normal. The bowels moved by aperients. The thoracic, abdominal organs, and urine were quite healthy. He was unable to stand, but could just move the legs slightly at knee and hip joints, otherwise they were paralysed. The paralysis was flaccid. There was no foot drop. The muscles were not wasted : they all reacted to a Faradic current. The constant current gave so much pain that it could not be used. There was no affection of sensation to pin, cotton wool, heat or cold. The sense of movement and of position was not lost.

*Reflexes.*—The deep reflexes in the legs were absent, in the arms absent or very feeble.

*Superficial reflexes.*—Plantar absent, cremasteric present, abdominal very feeble. No pain on passive movement of limbs. Slight loss of power in forearm and hands. Special senses and cranial nerves unaffected. No optic neuritis ; no alteration in speech or swallowing. Sphincters unaffected. Spinal column normal : no tenderness over spines. Cerebro-spinal fluid on lumbar puncture gave a slight excess of cells, lymphocytes and endothelial cells. Wassermann's test negative for blood and cerebro-spinal fluid. A swab from throat showed no Klebs-Löffler or diptheroid bacilli. Blood normal : no leucocytosis. Bacteriological examination of urine negative. Stools appeared healthy. At this stage it was impossible to make the diagnosis as to the exact nature of the lesion causing the slowly spreading paralysis of the legs. The mental condition was quite clear and good.

During the next two weeks he suffered much pain in the neck and back of the head. At the end of this time paralysis of both lower extremities had become almost complete, except for very slight movement at the hip-joints ; there was now marked foot-drop on both sides. Numbness (without objective loss of sensation) and loss of power in the hands had increased, each grasp registering only 15 on inner dynamometer scale, and the deep reflexes in the arms were now completely abolished. There was still no muscular wasting, and his condition was otherwise unchanged. A few days later the abdominal reflexes disappeared, and he complained much of a girdle pain. Three weeks after admission electrical reactions showed in the legs diminished reaction to interrupted current, especially in dorsiflexors of feet and long extensors of toes. To constant current the calf muscles gave normal reaction. The quadriceps extensors only reacted to a very strong current Right KCC=10 m.a. Left, KCC=14 m.a. ACC=18 m.a. Peronei and tibialis antici on both sides, KCC=1 m.a. ACC=2.5–3 m.a., and the contraction wave was slow.

On November 6th there were slight signs of wasting in the leg muscles, especially marked in the anterior muscles of the legs below the knee. The muscles were now also distinctly tender to pressure, and there was pain on passive movements of the ankles. There was a definite reaction of degeneration (R.D.) in peronei and tibialis antici muscles, and also some loss of sensation of movement and of position in left leg. Sensation otherwise unaffected.

By the end of November the paralysis had increased in the forearms and hands, which had become very weak, and the muscles of the forearms and the interossei appeared to be wasting. He could feed himself, but not cut up his food. There was indefinite and slight loss of tactile sensation on outer borders of feet. The only movement left in the lower extremities was internal rotation at hip-joints. The muscles of the lower extremities gave a slow, very feeble response to a very strong Faradic current ; those of the forearm, especially extensors, and of the hand also reacted badly, whilst the arm muscles gave a normal response. The nerves responded well. The leg muscles (below knee) now all showed reaction of degeneration to constant current. He remained much the same until the middle of January, when the forearms and hands began very slowly to gain power. The small muscles of the hands had wasted, and showed occasionally fibrillary twitchings. During this time all the muscles had become tender to pressure. The foot-drop had increased, and the legs were now placed in splints. Sensation remained normal, and the bladder unaffected. There was continued constipation. He could not sit up in bed. The diaphragm was never involved. All the reflexes in the limbs were lost, but the cremasteric and abdominal reflexes, though feeble, were present. The only muscles of the legs that responded now to the Faradic current were the right gastrocnemius and tibialis anticus, and adductors of left thigh.

During March he began slowly to improve, regaining first power in arms and hands, and then in hips. The muscles were no longer tender. In April he could sit up in a chair, swing his legs backwards and forwards, and slightly extend the left leg. There was, however, yet no recovery of power in muscles below the knee. In May he could very nearly stand without support, could walk with the aid of a walking machine, and by the end of the month could walk by pushing a chair in front of him. The foot-drop still remained for a long time afterwards. The subsequent course of the case has been one of very slow but steady recovery, so that he is now able to get about, and is going to take up some light employment. The knee-jerks are still absent.

In the following case, as in the preceding instance, the cause of the disease could not be ascertained, the onset was insidious, whilst the patient was apparently in perfect health. His occupation exposed him to extremes of heat and cold, and often involved heavy muscular work.

The distribution in this case is peculiar, and differs from the two just reported. The disease began in the upper extremities, with two days later weakness in legs, not proceeding to actual paralysis for three to four days longer. Moreover, in the legs the thigh muscles were those chiefly affected, and those below the knee at first escaped, to be involved later. The paralysis in all four extremities was, however, strictly symmetrical, and from the onset was in the arms of the peripheral type, extensor paralysis predominating, and the extensor muscles alone showed commencing reaction of degeneration (R.D.). In the lower extremities the quadriceps extensors are especially affected, and show more wasting and alteration of electrical reactions than any other muscles of the body. In spite of the somewhat unusual distribution of the muscular paralysis, there is no doubt of the case being one of toxic polyneuritis. As in the other cases, there is a complete absence of sensory affection, subjective or objective, apart from pains in right shoulder at the onset. This was the only fatal case.

Alfred T., aged 50, a marine fireman. He had never had any illness of any kind, and denied any venereal disease. He had six healthy children. He had been moderate as to alcohol, but smoked about one ounce of tobacco daily.

Three weeks before admission, whilst in perfectly good health, he had pains in right shoulder; on the following day he went to work, but felt weakness in both arms and hands; on the third day the weakness had so much increased in the arms that he was unable to work. By this time he felt some weakness of the lower extremities, which did not increase, and he was able to get up and sit about until three days later, when, on getting out of bed in the morning, his legs gave way and he fell to the ground. He remained in bed practically helpless for ten days longer, and was then admitted to hospital. There were no symptoms of constitutional disturbance, no pain after the first day; some tingling and feelings of pins and needles in forearms, hands, legs and feet. He had had some headache, the mind has been quite clear. Profuse sweats occurred at night, but no fever. Sphincters unaffected.

He did not come into contact with lead in his work.

On admission he was a well-built, healthy-looking, well-nourished man. No blue line on gums. Gums and teeth healthy. He lay in bed on his back, with arms across the abdomen. Except for some movements in the shoulder, chiefly due to pectorales, serratus magnus and trapezius muscles, the upper extremities were completely paralysed. When they were raised there was double wrist-drop. He could not sit up, turn, or move himself in bed. In the lower extremities slight rotation of hips and slight dragging up of legs by thigh muscles were possible, the ham-strings and extensors of legs were paralysed, but he could flex and extend ankles and toes freely but feebly; with this exception the lower extremities showed complete paralysis, could not be raised off the bed, and were useless.

The paralysis was of the flaccid type. Slight wasting of muscles was noted in the left quadriceps extensor, perhaps also in the right, in the extensors of wrists and fingers, and in the muscles of the thumbs and the abductor indicis. It was nowhere conspicuous, and the other muscles preserved their normal size.

There was no paralysis of any of the cranial nerves. Pupils equal, dilated, reacted to light and accommodation. Optic discs normal.

Speech and swallowing normal. Movements of head and neck unaffected.

The chest moved well in respiration, the movements of the diaphragm were perhaps a little feeble.

Sensation to touch, pin-prick, heat and cold everywhere normal. He retained the sense of position of his limbs; passive movements were not painful, and the muscles nowhere tender.

*Reflexes.*—Deep; biceps and triceps jerks present on both sides, wrist, knee and ankle jerks absent.

*Superficial.*—Abdominal absent, cremasteric present, both plantar present and flexor in type.

Bladder unaffected ; bowels constipated. Pulse 80, regular. Respiration 24.

Thoracic and abdominal organs normal. Urine 1026, no albumin, no sugar, loaded with urates.

Cerebo-spinal fluid under normal pressure and clear. Cells normal, a few lymphocytes only being present. Wassermann's test negative.

*Examination of Blood.*—Red cells 6,000,000 per c.m., hæmoglobin 90 per cent., leucocytes 19,000 per c.m., polymorphs 69 per cent., mononuclears 30 per cent., eosinophils 1 per cent.

One week after admission electrical reactions were as follows :—

*Interrupted current.*—Normal response in all muscles except the extensors of wrists and fingers and quadriceps extensor on each side, where a strong current was necessary, and to a less degree in small muscles of hands.

*Constant current.*—Right arm, in long flexors and extensors, KCC=ACC, 7 and 5 ma. respectively. Left arm, long extensors, wrist and fingers, KCC=8 ma., ACC=6 ma. Flexors, wrists and fingers, KCC=5 ma., 7 ACC=8 ma. Right abductor indicis, KCC=3 ma., ACC=7 ma. Left KCC=6 ma., ACC=7 ma. Legs, right quadriceps extensor, KCC=12 ma., ACC=10 ma. Left quadriceps extensor, KCC=18 ma., ACC=12 ma.

Other muscles gave normal reactions. The patient was very tolerant of both currents.

Ten days after admission the condition of the arms as regards paralysis was unchanged, wasting of the forearm muscles was more evident ; the diaphragm was slightly affected ; there appeared to be little if any power in the abdominal muscles, to which the constipation was partly attributable ; the thigh muscles were slightly more wasted ; and the paralysis had slowly spread to the legs, so that hardly any movement of feet and toes was possible, and the right foot was dropped. Plantar reflexes present, but very feeble. Other reflexes absent. In other respects his condition was unaltered. Sensation good everywhere.

During the next few days the action of the diaphragm became increasingly feeble, the respiration was quickened, the pulse became increasingly rapid and weak, the heart's action feeble, and he had difficulty in swallowing. The movements of the tongue and speech were unaffected, the facial and oculomotor nerves were not involved. He died of heart failure two weeks after admission. A *post-mortem* examination was not permitted.

The fourth case is that of a woman aged 33. She had been married about eighteen months, and before marriage was completely worn out by nursing her mother through a long illness. Her father died of diabetes. She had had no previous illness, and had always been temperate. The illness began with pains and loss of power in the legs two to three months before her confinement, which took place about three months before admission to hospital. For two months previous to confinement she lay in bed with her legs drawn up, and screamed if they were extended. Delivery was normal, no instruments being used. After it the paralysis spread to the arms. The pains in the legs began some time before she lost power in them. There had been no pains in the arms, but they felt dead or numb. The limbs rapidly wasted, and for two months before admission she had been helpless and unable to feed herself. From the fifth month of pregnancy and continuously since she had suffered from salivation.

On admission she was emaciated, there being no subcutaneous fat, and the muscles everywhere showed an extreme degree of wasting. She lay on her left side with her knees fully flexed, and thighs flexed on abdomen. The legs were contracted in that position, and she screamed when they were moved.

Appetite good. Tongue furred and tremulous. Pulse 120, respiration 20. Temperature normal. Bowels constipated. The teeth were very bad, many carious stumps, gums spongy, with pus around roots of teeth. No blue line on gums, no evidence of any source of lead poisoning.

The salivary glands were not enlarged: there was an almost constant flow of saliva. The thoracic and abdominal organs appeared to be normal. Urine normal. The pupils were unequal, but reacted well to light and accommodation. Optic discs normal. There was paresis of muscles of left side of face. Otherwise the cranial nerves were normal, and swallowing and speech unaffected.

She complained of some pain in the neck, but movements of head and neck were free.

*Arms.*—She could raise the arms, and flex, and extend the elbows partially. All the muscles of arms and forearms were much wasted. There was double wrist-drop, the small muscles of the hands were wasted, the thumbs and index fingers were in position of extension, the other fingers flexed. The left index finger could be flexed, and the thumbs could be partially opposed and adducted: otherwise paralysis was complete.

*Trunk.*—The abdominal muscles and those of the back were much wasted; she could not sit up.

*Legs.*—Glutei were wasted, and there was great wasting of all the muscles of the legs, especially below the knee, where the anterior tibial and peronei had practically gone, leaving a hollow. The ham-strings and calf muscles were relatively well preserved. There was complete double foot-drop, and all the toes were in position of flexion.

The only movements possible were feeble flexion of the hips, and slight movement of the legs. From contraction of tendons and adhesions in the joints neither hips nor knees could be passively moved beyond a right angle ; and such movements caused much pain.

None of the muscles were tender, and there was no tenderness over the course of the nerves.

*Electrical Reactions.*—To Faradism muscles of arms acted well ; those of forearm, especially extensors, feebly. Small muscles of hands reacted well ; the quadriceps extensor and ham-string muscles gave a weak reaction. The calf muscles contracted to a strong current. No reaction was obtained from the peroneal nerves, peronei, tibialis anticus and long extensors of toes. The constant current gave her great pain, and the reactions could not be well taken, but no reaction of degeneration was found save in the muscles of the anterior and outer aspect of legs.

The deep reflexes were absent, abdominal present, plantar absent. There was nowhere any affection of sensation to light touch, deep pressure, pin-prick, heat or cold. The sense of localisation of touch and of position of limbs was not lost. The sphincters were unaffected.

The patient's mental condition was childish, she cried very easily ; but there was no loss of memory or confusion of ideas.

The first point in treatment was to get the limbs into better positions. She was therefore anæsthetised, numerous firm adhesions in and about the hip, knee, and ankle joints were broken down, and the legs placed on straight splints. The upper extremities were also freely moved at all joints.

Subsequent treatment was by gentle massage and passive movements and Faradism, with maintenance of the limbs in suitable postures in order to prevent dragging on the paralysed muscles. The salivation gradually ceased.

After five months' treatment in hospital she had recovered sufficiently to return home.

This is an example of the somewhat rare though well-recognised form of multiple neuritis which occurs in pregnancy, or during the puerperium. If the onset is during pregnancy, the symptoms may become more marked after labour, which, as in this case, may take place normally. The disease appears especially in patients who suffer severely from the vomiting of pregnancy. In puerperal cases the cause is generally held to be a septic infection ; in those arising during pregnancy it is attributed to an intoxication related to the physiological processes of that condition, or by others, *e.g.* (Huber) to infection from a previous puerperium or from uterine disease (Oppenheim). In some cases the progress of paralysis is rapid, and the disease may take the form of Landry's paralysis. The possibility of alcohol as an additional factor in causation must be remembered. In this case there was no evidence of alcohol, but the patient was exhausted by long nursing before she became pregnant.

The prognosis in this form of neuritis is as a rule favourable.

The last case is one in which the chief symptom was inco-ordination, combined, as the disease went on, with a certain amount of definite paralysis, the latter being symmetrical and of the peripheral type. The presence of actual muscular weakness is important in the diagnosis from tabes dorsalis, because it is not a feature of this disease until, at any rate, a late stage is reached. The ataxy in this case reached an extreme degree, as marked as I have ever seen it in a bad case of tabes dorsalis, and was, indeed, so severe as to render the limbs useless. Moreover, it was attended by marked hypotonus, so that the joints and limbs could be placed in very abnormal positions without causing discomfort. The loss of the muscular sense and of that of position of limbs was also pronounced. Some doubt has been thrown on the existence of severe ataxia in multiple neuritis, it being held that errors in diagnosis have been made, and that the cases are really examples of tabes dorsalis, in which the association of disease of peripheral nerves with that of the posterior columns of the cord in tabes dorsalis, which is of frequent occurrence, is more than usually pronounced and extensive.

Dr. Judson Bury sums up his study of the question [1] by saying (1) that ataxia is rare in peripheral neuritis even when

[1] Allbutt and Rolleston's *System of Medicine*, 1910, vii, 449.

cutaneous and muscular sensibility are profoundly affected; (2) that it occurs occasionally when signs of muscular weakness and of diminished cutaneous sensibility are slight or absent.

Undoubtedly marked ataxia is a rare feature of multiple neuritis, and especially when, as in this case, there is no affection of common sensation. This is the only instance of the kind and degree that has come under my observation. In the diagnosis in this case I would lay stress on the complete absence of all signs of affection of the spinal cord, and on the presence of a definite amount of paralysis in the legs of the peripheral type, symmetrical, and accompanied by some muscular wasting, and on the recovery that took place in a comparatively short time. These are the essential points in the diagnosis of ataxia due to polyneuritis. One may also remark on the affection of the facial nerve, and its recovery after a short time. This is the cranial nerve which is most commonly affected in multiple neuritis.

Another feature was the presence in the wasted muscles of fibrillary tremors or twitchings, also an unusual symptom. This symptom was also present in the second case. It is to be expected that it should sometimes occur in these severe cases of multiple neuritis in which the whole of the lower motor neurone, including the anterior horn cell, is more or less affected. The cause in this case may either have been alcohol, in spite of his statement that he had been temperate for some years, or an infection through the urinary tract, as he had B. coli pyuria, or possibly the action of the latter upon nerves previously sensitised by excessive indulgence in alcohol.

John P., æt. 49, was admitted to the hospital in July, 1906, complaining of loss of power in the legs and arms. The onset was gradual, and began in the left leg a few months before: during the last month the arms and hands had become affected.

He was a traveller, and had had no previous illness except a chill (? influenza) twelve months before. For twenty years, up to the age of 42, he had been accustomed to drink about a bottle of whisky daily in the course of his business; since that age, according to his own statement, he had been a teetotaller. For some months he had been unable to get about, and had been confined to bed for over one month. He had never had syphilis.

He was a well-nourished man, with florid complexion and dilated venules on cheeks, and watery eyes, the vessels were thickened and tortuous; the pulse full and of high tension; the heart's apex beat was one inch outside the nipple line, the aortic second sound accentuated; the urine (sp. gr. 1010) showed no sugar or albumin; the lungs and abdominal organs appeared healthy.

There was no blue line on the gums; the tongue was tremulous but not wasted: its movements were inco-ordinated. He could not stand or sit up in bed, could not use his hands to feed himself, or hold a newspaper. When he tried to pick up anything the movements of the hand and arm had a peculiar swooping character. All movements of the hands, arms, legs and feet were wildly ataxic in character, and this was greatly increased when his eyes were closed. Without sight he had no idea of the position of his limbs. There was no absolute paralysis, but the extreme ataxy practically meant that he could not make use of his limbs for any purpose. Though no movements of the lower extremities were entirely absent, yet there was considerable loss of power in them, in addition to the ataxy, and this loss of power was chiefly in the muscles moving the ankle and foot (periphery of limb). This was not the case in the upper extremities, where the right grip measured 30 and the left 20 on inner scale of dynamometer. The muscles of the arms, hands, and trunk were not wasted. On the other hand, the glutei and all muscles of lower extremities, except the ham-strings, were wasted, and the wasting was especially marked in the anterior muscles of the thigh, and tibialis anticus and peronei group. The big toes were dropped, but not the feet.

There were irregular muscular twitchings almost amounting to fibrillary tremor in the most wasted muscles. The muscles all reacted to both interrupted and constant currents, but in the wasted muscles the response to the former was diminished, and to the latter increased, without any reversal of the normal formula. The muscles were flaccid, with loss of tone: in the legs there was considerable hypotony, it being possible to put the joints into very abnormal positions without causing pain. The muscles were not tender, but there was slight tenderness over nerve trunks.

*Reflexes.*—Superficial: abdominal and cremasteric present, plantar doubtful. The deep reflexes were absent in both arms and legs.

Sensation to light touch (cotton wool), pain (pin-prick), heat and cold was everywhere unaffected. Localisation of sensation was also good. The sphincters were unaffected throughout. Pupils equal, reacted to light, accommodation and consensually. Optic discs normal. Other cranial nerves normal. Special senses normal.

During September he had two rigors and fever, with the appearance of a quantity of pus in the urine due to a B. coli infection of urinary tract. The symptoms cleared up under treatment in the course of a month, but B. coli with a few pus cells persisted in the urine. After the first rigor there appeared partial paralysis of the right facial nerve, which gradually disappeared during the next fortnight.

The general treatment was by massage, electric baths, and systematic training in movements of the limbs. For a time the wasting of the leg muscles continued, and to such a degree that the calf muscles had the feeling described by Gowers of " a bag of worms." Fibrillary twitching in the muscles most wasted was often noticed. At the end of September he began slowly to improve, the arm muscles first recovered their tone, the movements became less inco-ordinate, and he was able to use the arms and hands. Improvement in the legs followed more slowly, the fibrillary twitchings ceased, movements improved, and the wasted muscles very gradually recovered. By December he was able to stand and walk with support, and in January, 1907, with the aid of sticks. Though not recovered, for recovery of the wasted muscles would take a still longer time, he was so much improved and so much more able to help himself, that he left the hospital in this month. The deep reflexes were still entirely absent. There were no mental changes throughout the illness. There were no shooting pains, no joint or skin lesions, and no tendency to bed sores.

